# Respiratory Syncytial Virus Activates Rab5a To Suppress IRF1-Dependent Lambda Interferon Production, Subverting the Antiviral Defense of Airway Epithelial Cells

**DOI:** 10.1128/JVI.02333-20

**Published:** 2021-03-25

**Authors:** Shi Mo, Wei Tang, Jun Xie, Sisi Chen, Luo Ren, Na Zang, Xiaohong Xie, Yu Deng, Leiqiong Gao, Enmei Liu

**Affiliations:** a Department of Respiratory Medicine, Children’s Hospital of Chongqing Medical University, Chongqing, People’s Republic of China; b National Clinical Research Center for Child Health and Disorders, Chongqing, People’s Republic of China; c Ministry of Education Key Laboratory of Child Development and Disorders, Chongqing, People’s Republic of China; d Chongqing Key Laboratory of Child Infection and Immunity, Chongqing, People’s Republic of China; Hudson Institute of Medical Research

**Keywords:** respiratory syncytial virus, Ras-related protein in brain 5a, interferon regulatory factor 1, interferon lambda, antiviral defense

## Abstract

The limited antiviral options and lack of an effective vaccine against human respiratory syncytial virus (RSV) highlight the need for a novel antiviral therapy. One alternative is to identify and target the host factors required for viral infection. Here, using RNA interference to knock down Rab proteins, we provide multiple lines of evidence that Rab5a is required for RSV infection: (i) Rab5a is upregulated both in RSV A2-infected A549 cells and RSV A2-challenged BALB/c mouse airway epithelial cells at early infection phase; (ii) short hairpin RNA (shRNA)-mediated knockdown of Rab5a is associated with reduced lung pathology in RSV A2-challenged mice; (iii) Rab5a expression is correlated with disease severity of RSV infection of infants. Knockdown of Rab5a increases lambda interferon (IFN-λ) production by mediating interferon regulatory factor 1 (IRF1) nuclear translocation. Our results highlight a new role for Rab5a in RSV infection, such that its depletion inhibits RSV infection by stimulating the endogenous respiratory epithelial antiviral immunity, which suggests that Rab5a is a potential target for novel therapeutics against RSV infection.

**IMPORTANCE** This study highlights the important role of Rab5a in respiratory syncytial virus (RSV) infection, such that its depletion inhibits RSV infection by stimulating the endogenous respiratory epithelial antiviral immunity and attenuates inflammation of the airway, which suggests that Rab5a is a powerful potential target for novel therapeutics against RSV infection.

## Abstract (precis)

This study highlights the important role of Rab5a in respiratory syncytial virus (RSV) infection, such that its depletion inhibits RSV infection by stimulating the endogenous respiratory epithelial antiviral immunity and attenuates inflammation of the airway, which suggests that Rab5a is a powerful potential target for novel therapeutics against RSV infection.

## INTRODUCTION

Human respiratory syncytial virus (RSV) belongs to the *Pneumoviridae* family (1) and is a leading cause of respiratory tract infection in young children. Approximately 4 million children worldwide are admitted to hospitals each year with RSV infection, 3.4 million of whom develop severe symptoms such as bronchiolitis and pneumonia (2–4). The health care costs of hospitalization from RSV-infected patients are significant (5, 6), and despite years of ongoing efforts, there is currently no safe or effective vaccine available to protect the children from RSV and minimize the global burden. Thus, identification of host factors required for RSV infection may constitute a plausible alternative to develop a therapeutic regimen.

Airway epithelial cells are the target cell types for RSV infection. Being obligatory intracellular parasites, viruses utilize diverse cellular trafficking machinery to achieve productive life cycles in the infected host cells. Members of the Rab family of cellular proteins regulate actin- or microtubule-based motor proteins and intracellular membrane trafficking and have been implicated in various steps of the viral life cycle, including replication, assembly, and budding (7, 8). In addition, the Rab family proteins are involved in innate immunity (9). In the present study, in order to identify cellular Rab proteins required for RSV infection, we interrogated the role of nine widely expressed Rab proteins (Rab1a, Rab2a, Rab4a, Rab5a, Rab6a, Rab7a, Rab8a, Rab9a, and Rab11a) that are involved in the endo- or exocytic pathways. Using specific small interfering RNA (siRNA) to knock down each Rab protein, we found that the micropinocytosis-associated Rab5a protein is required for RSV infection.

Rab5a plays a critical role in viral infection (10–12). The involvement of Rab5a in RSV endocytosis or micropinocytosis has been described previously (13). In parallel, several studies demonstrated that Rab5a is closely related to innate immunity, notably, the interferon (IFN)-signaling JAK-STAT pathway, and downregulation of Rab5a was shown to increase STAT1 expression (14, 15). Rab5a is also required for the formation of the early endosome, which is related to the IFN-induced transmembrane proteins of the IFITM family; moreover, the type I IFN receptor complex is differentially sorted at the early endosome (16). Taken together, these studies suggest that Rab5a may affect the innate immunity in RSV infection. Lastly, several RNA viral nonstructural (NS) proteins, such as the NS proteins of RSV, subvert IFNs, and Rab5 has been shown to colocalize with NS-induced structures of the Severe fever with thrombocytopenia syndrome (SFTS) virus (17). Based on these findings, we hypothesize that Rab5a facilitates RSV infection through the inhibition of the cell-intrinsic antiviral IFN pathway.

As mentioned, IFN signaling is a major arm of the innate antiviral response of the host. Recent studies revealed that IFN-λ, a type III IFN, is also an important IFN of the airway epithelium (18, 19). Further studies suggested that type I IFNs (i.e., IFN-α and IFN-β) are critical for the clearance of infection, whereas IFN-λ is the most important IFN regulating mucosal epithelial cell responses to viral infection (19, 20). Recent studies found that IFN-λ is the first produced IFN of the RSV-exposed nasal epithelium (21). Moreover, RSV could inhibit IFN-λ production in lung epithelial cells, and IFN-λ was critical for antiviral immunity to RSV (22, 23). Further studies suggested that RSV-induced epidermal growth factor receptor (EGFR) activation suppresses IFN-λ production by inhibiting interferon regulatory factor 1 (IRF1), a transcription factor for the IFN-λ gene (21). However, the potential role of the Rab5a pathway in modulating IFN-λ and its related innate immunity in RSV infection has not been reported. Here, we have explored the effect of the Rab5a pathway on RSV infection in airway epithelial cells and the role of IFN-λ-related factors in this process. We found that the small GTPase Rab5a, which is exclusively upregulated in RSV-infected airway epithelial cells, inhibits host responses against RSV infection by regulating the pathways related to IFN-λ. Our data thus demonstrated that inducible Rab5a serves as an inhibitor to control IFN-λ-mediated antiviral defense in airway epithelium.

## RESULTS

### Rab5a is essential for RSV production.

**FIG 1:**
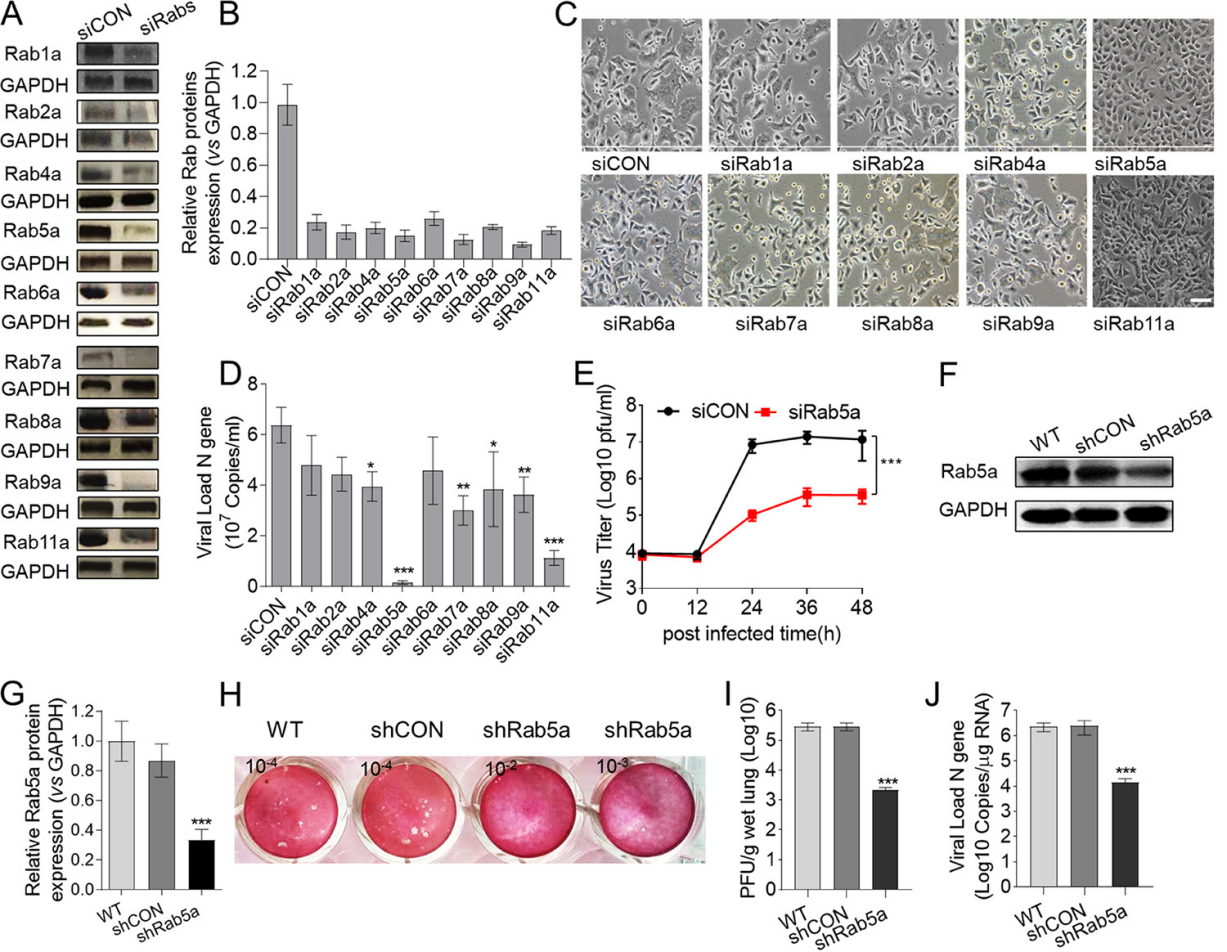
Rab5a depletion reduces RSV propagation. A549 cells were transfected with either siRNA control (siCON) or specific siRNAs targeting Rab proteins (siRabs) and then infected with RSV (MOI = 0.8) as described in Materials and Methods. (A) Depletion of Rab proteins was confirmed by immunoblotting analysis of the total cell lysates. (B) Semiquantitative analysis of the data from panel A, using Image J. (C) Bright-field images of infected cell cultures, taken at 36 h p.i. Bar, 20 μm. (D) RSV propagation was scored by measuring the amount of RSV both in attached cells and released into the culture supernatants by qPCR of the RSV N gene at 36 h p.i. (E) Time course of RSV propagation at an MOI of 0.8. Female BALB/c mice, 6 to 8 weeks old, were intranasally infected with lentivirus-packaged shRNA control (shCON) or specific shRNA targeting Rab5a (shRab5a) three times and then infected with RSV as described in Materials and Methods. (F) Depletion of Rab5a was confirmed by immunoblotting analysis of the total lung lysates. (G) Semiquantitative analysis of the data from panel F, using ImageJ. (H and I) Viral titers were measured by plaque assay. (J) RSV propagation was scored by measuring the amount of RSV in lung tissue by qPCR; the values are shown as means ± standard deviations (SDs) (*n* =4 to 8/group). Differences between siCON and siRab5a were analyzed for each time point by multiple *t* tests according to the Holm-Sidak method. ***, *P* < 0.0001 (for each time point from 24 h through 48 h p.i.). The error bars indicate SDs. The other *P* values were obtained by one-way ANOVA with a Bonferroni posttest. ***, *P* < 0.0001; **, *P* < 0.001; *, *P* < 0.05.

To assess the effect of the Rab family on RSV replication, we depleted Rab1a, Rab2a, Rab4a, Rab5a, Rab6a, Rab7a, Rab8a, Rab9a, and Rab11a by treating A549 cells with specific siRNAs (or control siRNA) for 24 h, followed by infection with purified RSV A2 and incubation for another 36 h. Depletion of Rab proteins was confirmed by immunoblotting analysis of the total cell lysates (Fig. 1A and B). The syncytium morphology of RSV is shown in Fig. 1C. To determine the viral load, the viral N gene RNA was measured by quantitative PCR (qPCR) on RNA isolated at 36 h postinfection (p.i.) (Fig. 1D). Little alteration of RSV replication was observed following depletion of Rab1a, Rab2a, and Rab6a compared to that in the cells treated with control siRNA (siCON). A moderate decrease in RSV replication was observed upon knockdown of Rab4a, Rab7a, Rab8a, and Rab9a, with 40% to 70% less N gene detected in both the cells and supernatant compared to that in the control. The effects of Rab11a depletion were similar to those reported previously (24, 25). The most potent effect on RSV propagation was observed in cells depleted of Rab5a (Fig. 1C and D), and the level of N was >30-fold lower than in the control (Fig. 1D). We further measured the growth kinetics of RSV in siCON- and siRab5a-treated cells and found that knockdown of Rab5a decreased RSV replication (Fig. 1E). These results suggested that Rab5a plays an important role in RSV propagation.

To further assess the effect of Rab5a on RSV infection *in vivo*, Rab5a was knocked down using specific short hairpin RNA (shRNA) lentivirus or control and the mice were infected with RSV A2 virus. Rab5a knockdown efficiency by shRNA was confirmed by immunoblotting (Fig. 1F and G). Plaque assay revealed that Rab5a knockdown resulted in lower viral titer than the control at 3 days p.i. (Fig. 1H and I). The viral load data displayed the same trend (Fig. 1J) as the plaque assay results. Taken together, Rab5a is essential for RSV propagation.

### Rab5a is upregulated in RSV-infected respiratory epithelium.

**FIG 2:**
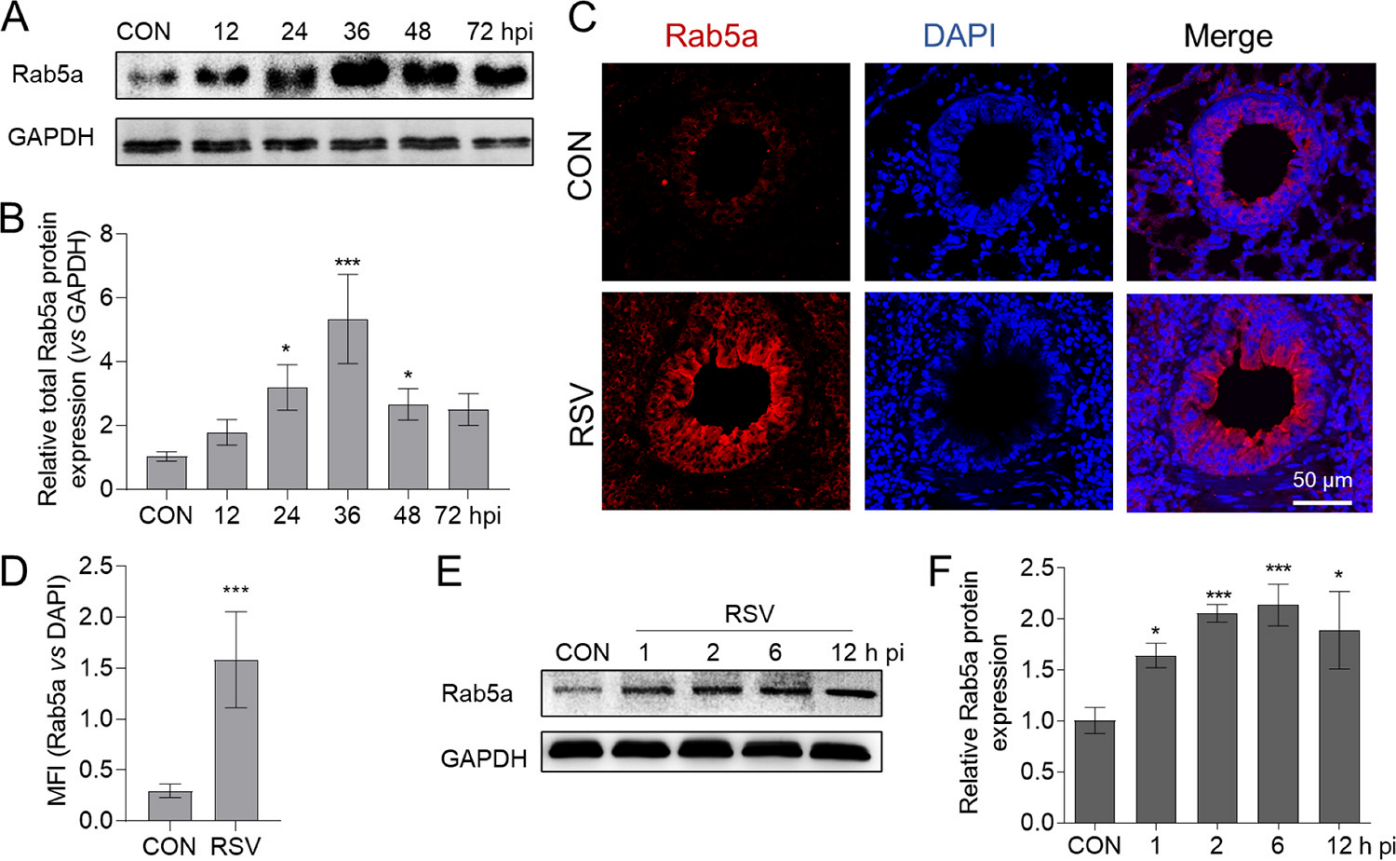
RSV infection upregulates Rab5a expression at early infection times. Intranasal delivery of lentiviral shRNA against Rab5a and infection with RSV A2 were conducted as described for Fig. 1. At the indicated times, mice were sacrificed to measure the lung’s Rab5a protein expression by immunoblotting (A) and immunofluorescence (C) at 36 h p.i. (B) Semiquantitative analysis of the data for panel A, using ImageJ. (D) Mean fluorescence intensity analysis of tracheal ring epithelial cell data from panel C. Bars represent means ± SDs (*n* = 4 to 8/group). A549 cells were infected with RSV, and at the indicated times (1, 2, 6, and 12 h p.i.), samples were collected for measurement of Rab5a protein. (E) Rab5a protein expression at various postinfection times. (F) Semiquantitative analysis of the data from panel E, using ImageJ. Bars represent means ± SDs from three independent experiments performed in duplicates. *P* values were calculated based on unpaired Student’s *t* tests between control and RSV infection in panel D. *P* values in panels B and were obtained by one-way ANOVA with a Bonferroni posttest. ***, *P* < 0.0001; *, *P* < 0.05.

To investigate the dynamic Rab5a expression in RSV-infected BALB/c mice, we first measured the Rab5a protein level at different times of infection. Increase in total Rab5a protein level (Fig. 2A and B) was detected at 12 h p.i., which peaked at 36 h p.i. The immunofluorescent antibody (IFA) staining of Rab5a further displayed that Rab5a was mainly upregulated in the respiratory epithelial cells (Fig. 2C and D). Then, we next assessed dynamic Rab5a expression in RSV-infected A549 cells. It was observed that Rab5a protein levels significantly increased starting at 1 h p.i. (Fig. 2E and F) compared with that for mock infection. These results confirmed that RSV infection upregulated the respiratory epithelium’s Rab5a expression.

### Knockdown of Rab5a does not alter virus binding and endocytosis in the epithelium.

**FIG 3:**
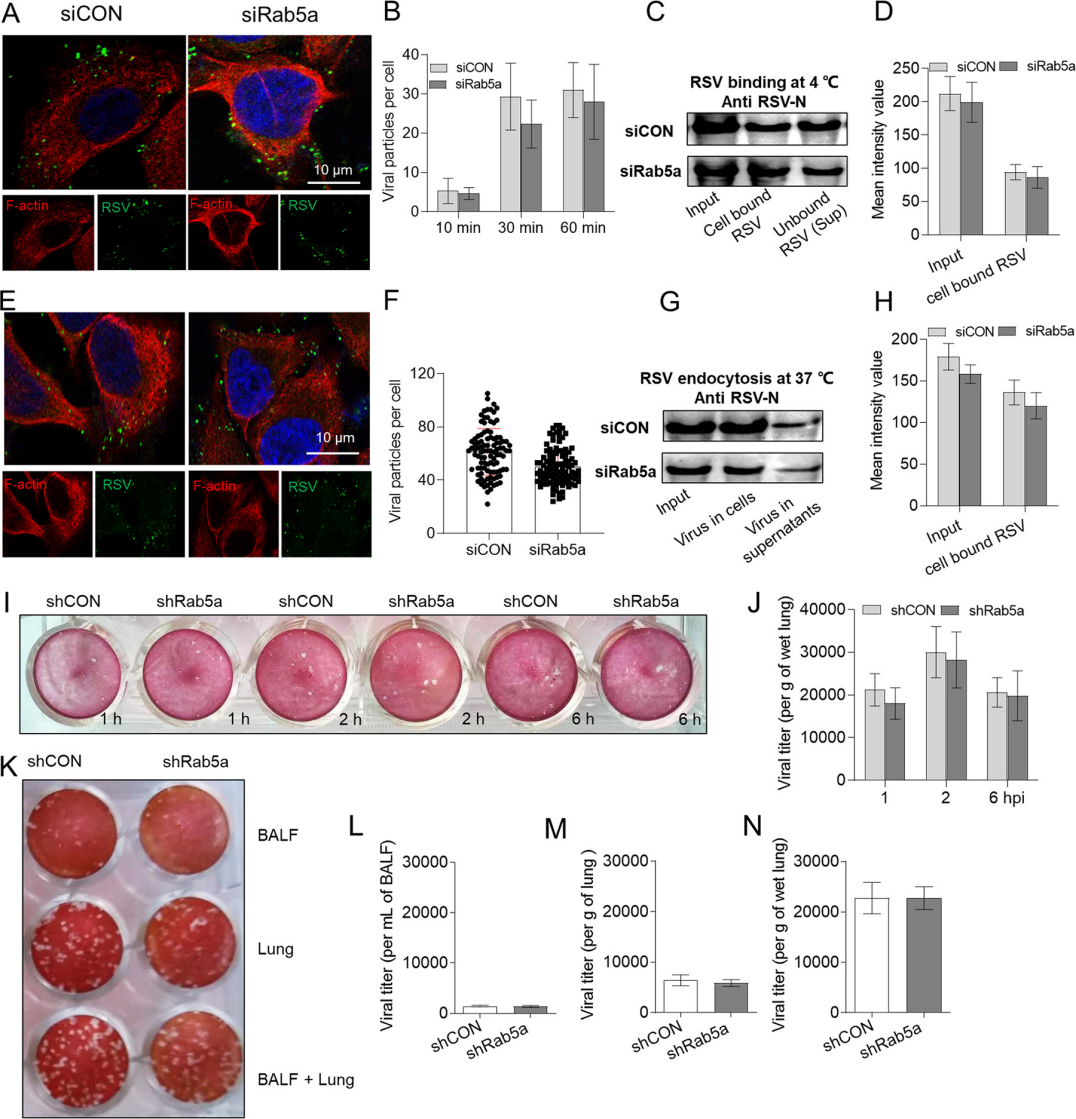
Knockdown of Rab5a does not alter virus binding and endocytosis in epithelial cells. A549 cells were incubated with equal amounts of RSV (MOI = 10) at 4°C. The cells and medium supernatants were collected at the indicated times as described in Materials and Methods. The cells were fixed and stained with F-actin and RSV N antibodies. (A) Single-focal-plane images of cell samples were acquired with a confocal microscope. (B) The numbers of green virus particles were estimated from 100 cells by using ImageJ. (C) The cell-bound virus lysates (bound), supernatants (unbound virus), and input virus were resolved by SDS-PAGE followed by immunoblotting with an RSV N antibody. (D) Semiquantitative analysis of the data from panel C, using ImageJ. (E) A549 cells were incubated with RSV (MOI = 10) at 4°C for 1 h, unbound virus was removed, and cells were incubated at 37°C for 1 h before the supernatant and the cells were collected. Cells were fixed and photographed (E), and the numbers of green viral particles in 100 cells were measured with ImageJ (F). (G) Input virus, cell lysis, and the supernatant virus were analyzed by SDS-PAGE, and then immunoblotting was performed with an RSV N antibody. (H) Semiquantitative analysis of the data from panel G, using ImageJ. Bars represent means ± SDs from three independent experiments performed in duplicates. Female BALB/c mice, 6 to 8 weeks old, were intranasally infected three times with either lentivirus-packaged shRNA control (shCON) or specific shRNA targeting Rab5a (shRab5a) and then infected with RSV A2 (1.5 × 10^7^ PFU/mouse). (I and J) At the indicated times, viral titers of whole-lung tissues were quantified by plaque assay. (K to N) At 6 h p.i., BALF (unbound virus) was collected, and the lung tissue (bound virus), whole lung (unbound virus and bound virus), and viral titers of cells were quantified by plaque assay. Bars represent means ± SDs (*n* = 4 to 8/group). *P* values were calculated based on unpaired Student’s *t* tests between siCON or shCON and siRab5a or shRab5a.

Because the Rab5a expression level was increased at an early infection time and viral and host factors are involved in early infection, we assessed the effect of Rab5a on viral early life cycle procedures (viral binding and endocytosis) in RSV infection of A549 cells. For viral binding, there was no significant difference between siRab5a and control groups (Fig. 3A to D). For viral endocytosis, there was also no difference between siRab5a and control groups (Fig. 3E to H).

To confirm these results *in vivo* in mice, 6- to 8-week-old female mice were infected with RSV A2. We first measured the viral titers in lung tissue without performing bronchoalveolar lavage and found there was no significant difference in viral loads between Rab5a knockdown and control groups at various postinfection times (1, 2, and 6 h p.i.) (Fig. 3I and J). Furthermore, we also tested the viral loads in whole lungs without performing bronchoalveolar lavage, in bronchoalveolar lavage fluids (BALF), and in the lung tissues after performing alveolar lavage at 6 h p.i. and found there was also no significant difference between Rab5a knockdown and control groups (Fig. 3K to N). Taken together, these data suggested that Rab5a has no effect on viral binding and endocytosis.

### Knockdown of Rab5a increases IFN-**λ** production.

**FIG 4:**
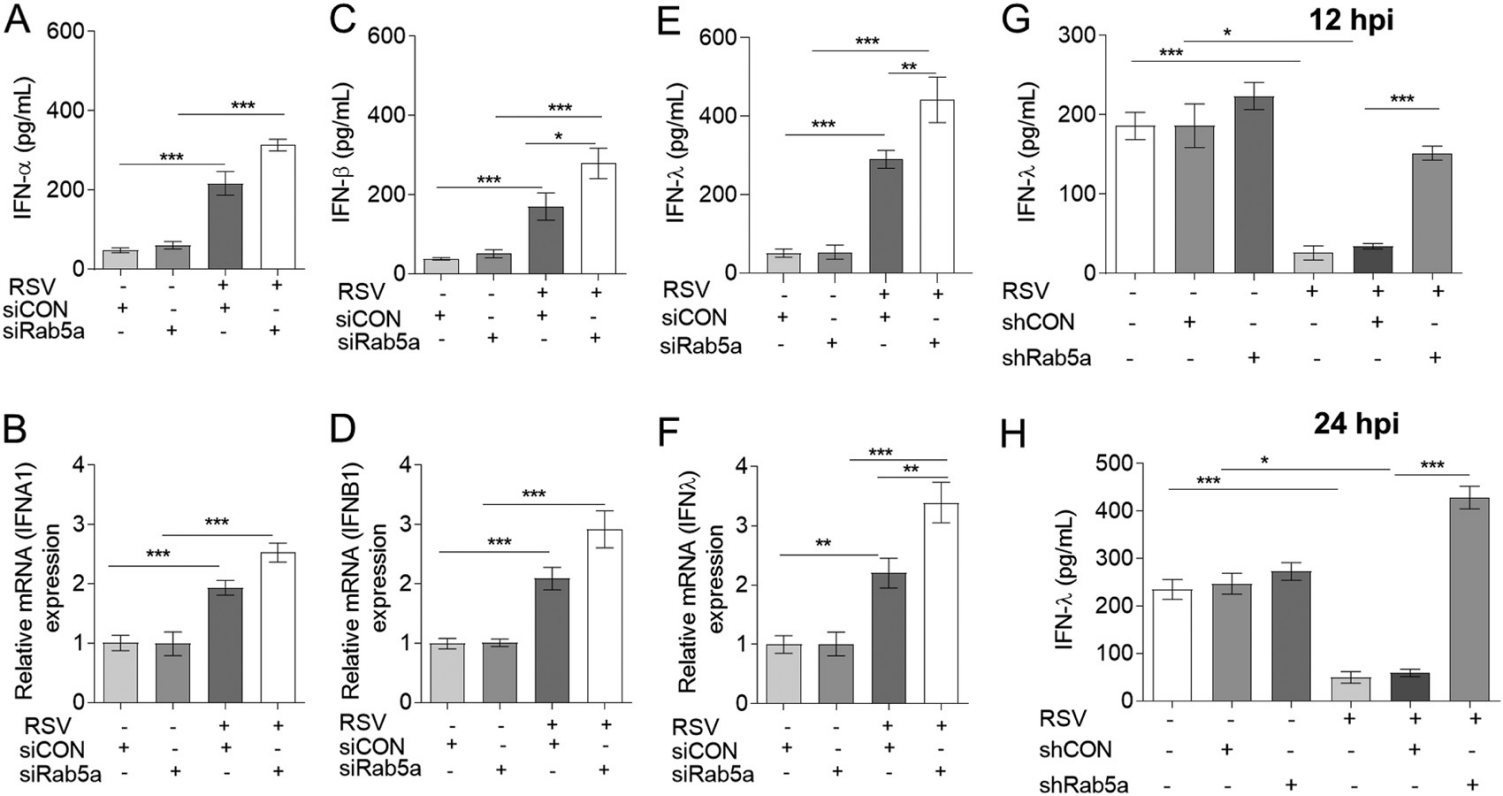
Knockdown of Rab5a increases IFN-λ production. Transfection of A549 cells with siRNA control (siCON) or Rab5a siRNA, followed by infection with RSV, was carried out as described in Materials and Methods. All mRNAs were quantified by qPCR, and IFN in the supernatant was quantified by ELISA. (A) IFN-α; (B) IFN-α (IFNA1) mRNA; (C) IFN-β; (D) IFN-β (IFNB1) mRNA; (E) IFN-λ; (F) IFN-λ mRNA. Bars represent means ± SDs from three independent experiments performed in duplicates. Nasal instillations of shRNA control (shCON) or Rab5a shRNA lentivirus, followed by infection with RSV, are described in Materials and Methods. At 12 h and 24 h p.i., IFN-λ in bronchoalveolar lavage fluid was measured by ELISA. Bars represent means ± standard errors (SEs) (*n* = 8 to 10/group). *P* values were calculated based on one-way ANOVA with Bonferroni posttest. ***, *P* < 0.0001; **, *P* < 0.001; *, *P* < 0.05.

All of the above-described data demonstrated that Rab5a subverts another mechanism to promote RSV growth. At an early infection time, the airway epithelial cells’ innate immunity plays an important role against viral infection. Type I and type III IFNs play important roles in innate and adaptive antiviral immunity. As indicated earlier, recent studies have implicated that besides IFN-α and IFN-β, IFN-λ is another important IFN that responds to RSV infection in the respiratory epithelia. We therefore focused on the potential role for Rab5a in regulating the production of all three IFNs. A549 cells were transfected with siRNAs specific for Rab5a (and siRNA control) for 24 h and were then infected with RSV for another 24 h. Control mock-infected cells received the same volume of medium. Results showed that RSV increased IFN-α (Fig. 4A and B), IFN-β (Fig. 4C and D), and IFN-λ (Fig. 4E and F) expression and production compared with that for mock infection. Rab5a knockdown further increased IFN-λ production significantly (Fig. 4E and F) but had no effect on IFN-α and IFN-β production compared to that for siCON in RSV-infected cells (Fig. 4A to D). We next measured IFN-λ production in bronchoalveolar lavage fluid from RSV-infected BALB/c mice. We also found that knockdown of Rab5a enhanced IFN-λ production compared to that in the controls at an early infection phase (12 and 24 h p.i.) (Fig. 4G and H). These data suggest that knockdown of Rab5a increased IFN-λ production during RSV infection.

### Knockdown of Rab5a increases IRF1 expression and promotes its nuclear translocation.

**FIG 5:**
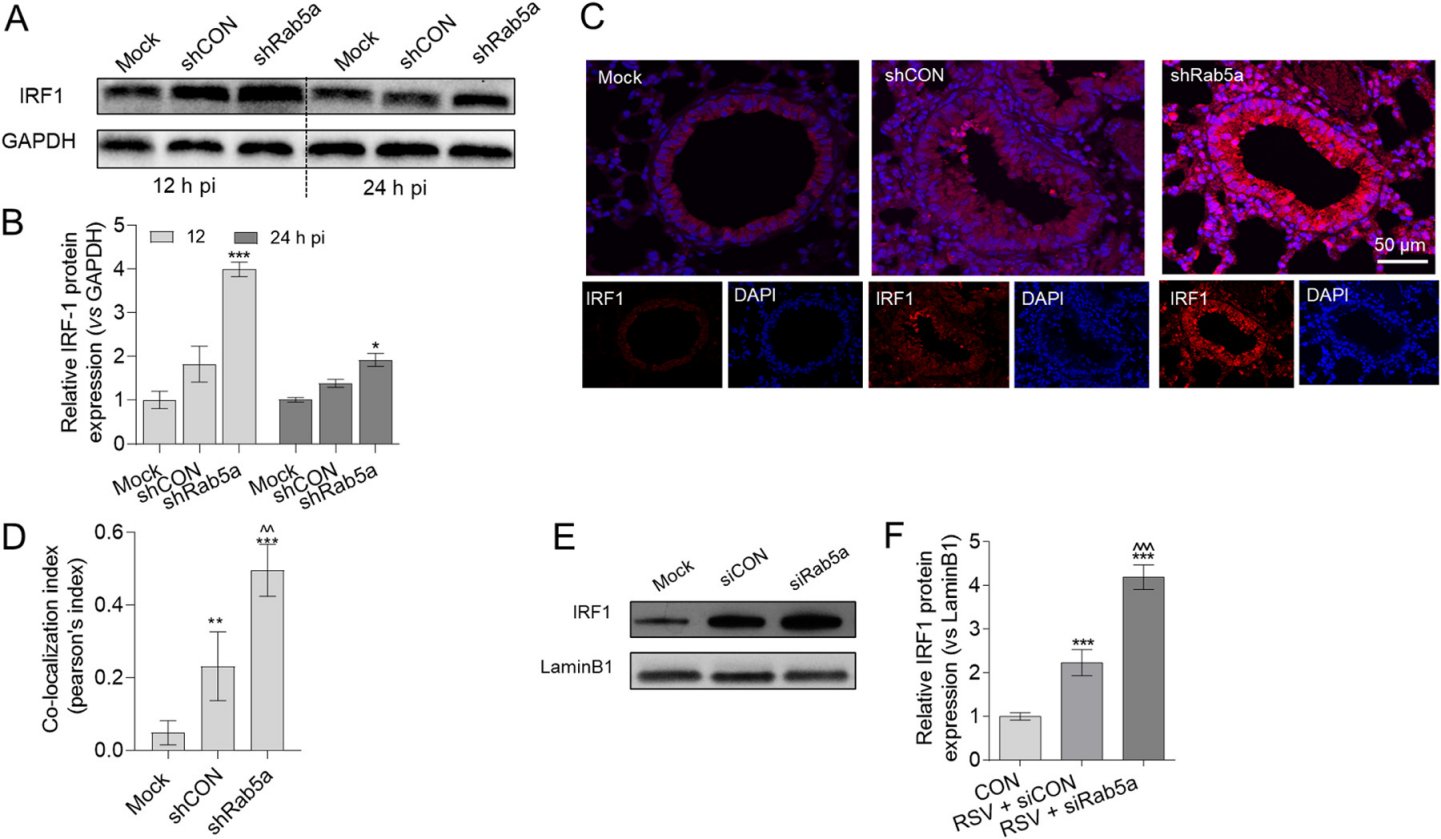
Knockdown of Rab5a exaggerates IRF1 production. Intranasal delivery of lentiviral shRNA against Rab5a and infection with RSV A2 were conducted as described for Fig. 1 (A) Immunoblotting to measure total IRF1 expression in the lung tissue at 12 or 24 h p.i. (B) Semiquantitative analysis of data from panel A, using ImageJ. (C) At 24 h p.i., single-focal-plane images of airway tissue samples with IFA staining were acquired with a confocal microscope. (D) Airway epithelial cells: DAPI and IRF1 colocalization was analyzed from images for panel C using NIS confocal software 4.1. Higher values represent greater colocalization. The data are representative of two independent experiments (*n* = 3 to 4 mice per experiment). A549 cells were transfected with either siCON or siRab5a for 24 h and then infected with RSV as described. (E) IRF1 protein expression, detected by immunoblotting. (F) Semiquantitative analysis of data for panel E, using ImageJ. Bars represent means ± SDs from three independent experiments performed in duplicates. *P* values were calculated based on one-way ANOVA with Bonferroni posttest. ***, *P* < 0.0001; **, *P* < 0.001; *, *P* < 0.05 for CON versus RSV. ^^^, *P* < 0.0001; ^^, *P* < 0.001 for siCON versus siRab5a.

The IFN regulatory factors (IRFs), functioning as transcription factors, play an important role in IFN production. RSV can activate IRF1 in monocytes and lung epithelial cells (26), and IRF1 can interact with the IFN-λ promoter to induce IFN-λ transcription. Thus, we next explored the effect of Rab5a on IRF1 expression during RSV infection. Rab5a was knocked down by lentivirus-packaged Rab5a shRNA in BALB/c mice, which were then infected with RSV for 12 h or 24 h. We found that RSV infection increased IRF1 levels, and knockdown of Rab5a further increased IRF1 expression during RSV infection (Fig. 5A and B) in whole-lung tissue. Immunofluorescence staining showed that IRF1 increased in airway epithelial cells; confocal laser scanning microscopy further showed that the nuclear translocation of IRF1 increased (Fig. 5C and D). Then, we transfected siRab5a and siCON into A549 cells for 24 h, and then the cells were infected with RSV for another 24 h. Western blotting (WB) and immunofluorescence staining confirmed that knockdown of Rab5a resulted in increased nuclear translocation of IRF1 (Fig. 5E and F). Overall, these results suggested that knockdown of Rab5a increased IRF1 expression and promoted its nuclear translocation.

### Knockdown of Rab5a increases IFN-**λ** production via active IRF1.

**FIG 6:**
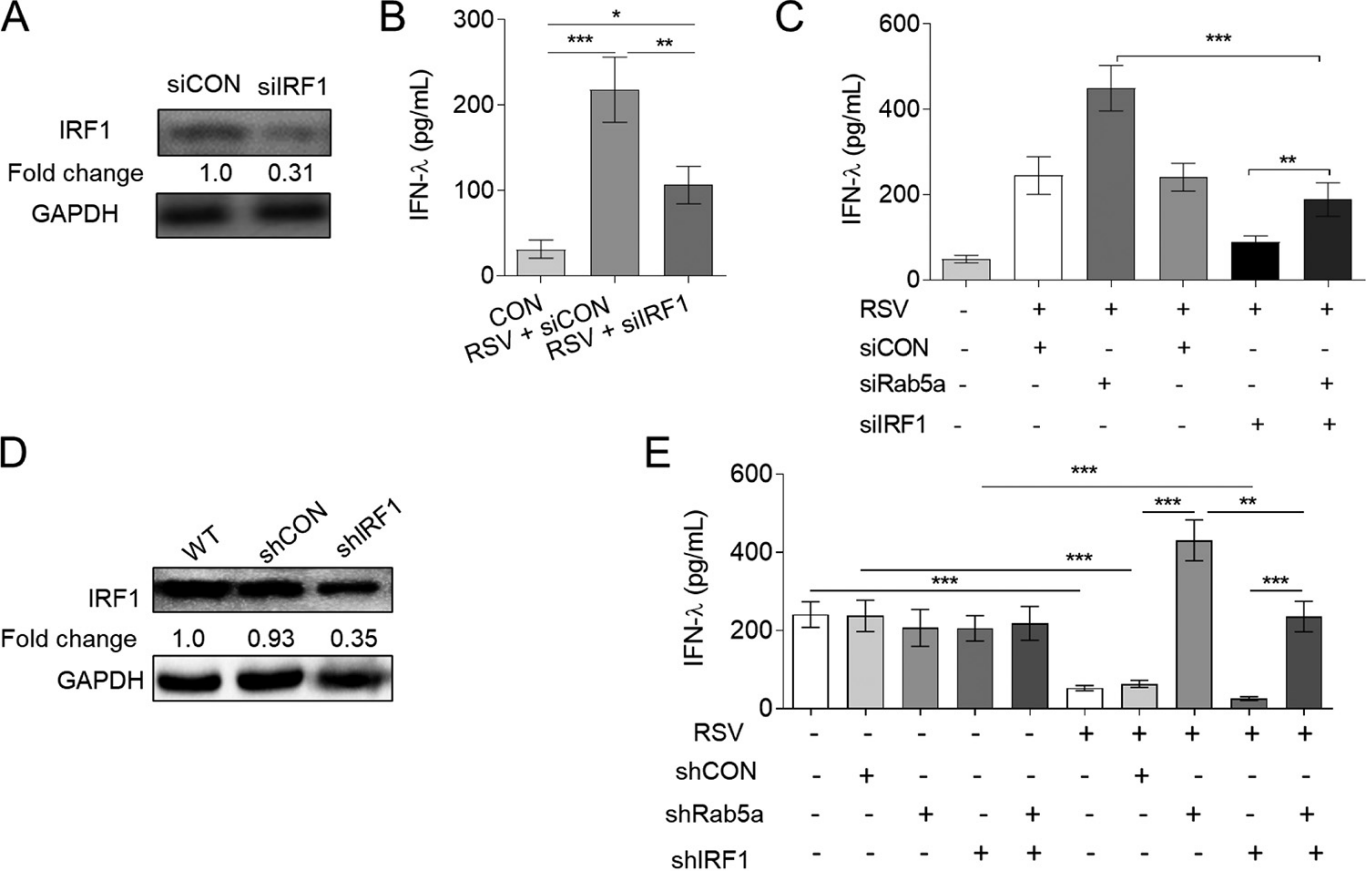
Rab5a mediates IFN-λ production via IRF1. A549 cell transfection with siRNA control (siCON) or siRNAs targeting IRF1 (siIRF1) and RSV infection were conducted as described in Materials and Methods. (A) Knockdown efficiency of IRF1 by specific siRNA, determined by immunoblotting. (B) IFN-λ detection in supernatants, using ELISA. Intranasal delivery of lentiviral shRNA against Rab5a or IRF1 (or control shRNA) and infection with RSV A2 were conducted as described for Fig. 1. Female BALB/c mice, 6 to 8 weeks old, were nasally instilled three times with either lentivirus-packaged shRNA control (shCON) or specific shRNA targeting IRF1 (shIRF1) or Rab5a (shRab5a). (D) Knockdown efficiency of IRF1 by specific shRNA determined by immunoblotting. (E) These mice were infected with RSV A2 (1.5 × 10^7^ PFU/mouse) for 24 h, and IFN-λ production was measured in bronchoalveolar lavage fluid, using ELISA. Bars represent means ± SEs (*n* = 8 to 10/group). *P* values were calculated based on one-way ANOVA with Bonferroni posttest. ***, *P* < 0.0001; **, *P* < 0.001; *, *P* < 0.05.

To confirm the important role of IRF1 in RSV-induced IFN-λ production in epithelial cells, we treated A549 cells with IRF1-specific siRNA, which significantly suppressed IRF1 protein levels (Fig. 6A). Treatment with this siIRF1 decreased IFN-λ production in RSV-infected A549 cells compared with that in cells infected with RSV but treated with control siRNA (Fig. 6B). Moreover, siIRF1 eliminated the effect of siRab5a in exaggerating IFN-λ production (Fig. 6C) that we showed earlier. We also confirmed these data in RSV-infected BALB/c mice at 24 h p.i. (Fig. 6D and E). Together, these results suggest that (i) depletion of IRF1 reduces IFN-λ production and (ii) siRab5a exaggerates IFN-λ production via IRF1.

### Knockdown of Rab5a alleviates airway inflammation from RSV infection.

**FIG 7:**
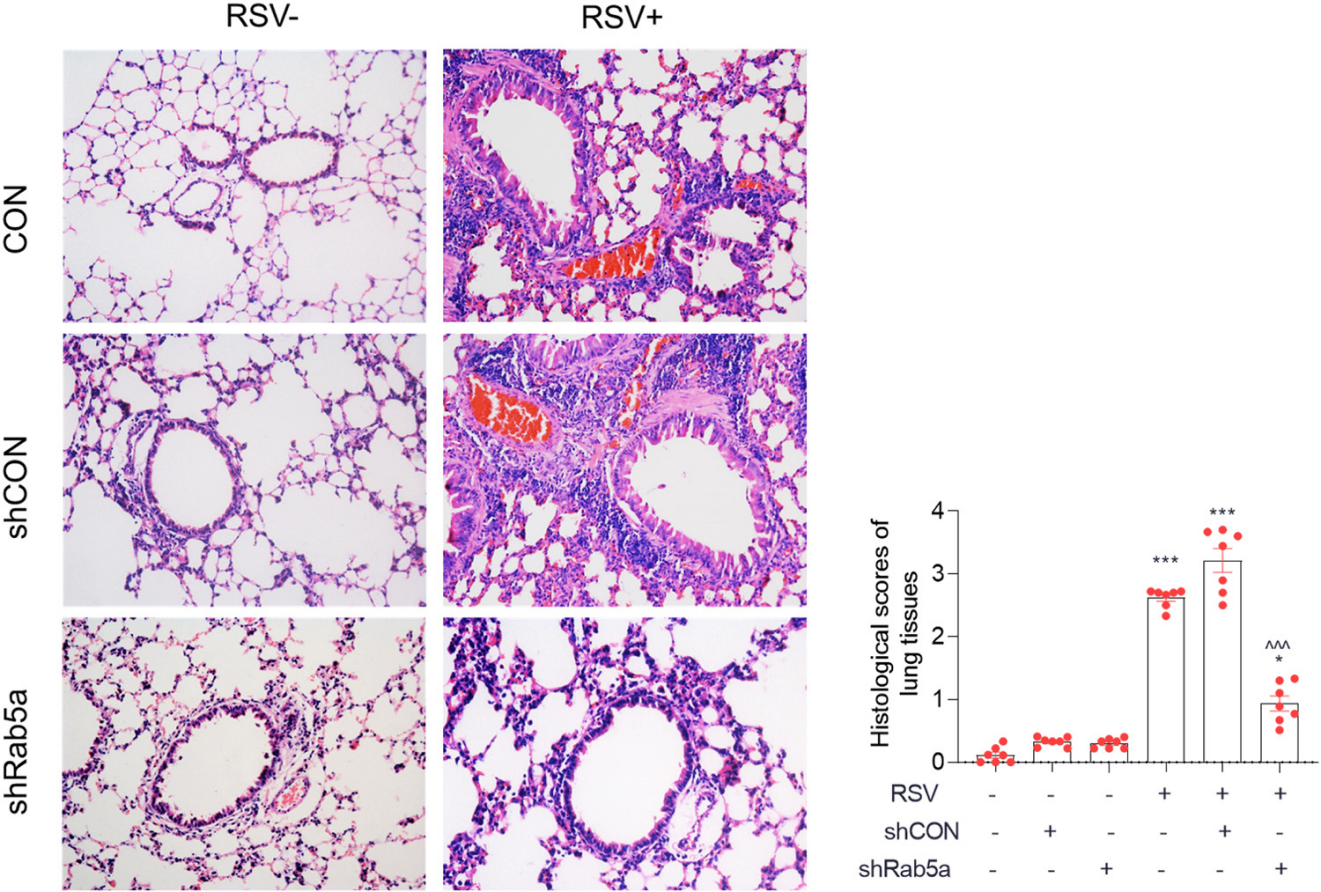
Knockdown of Rab5a alleviates airway inflammation during RSV infection. Intranasal delivery of lentiviral shRNA against Rab5a or control shRNA and infection with RSV A2 were conducted as described for Fig. 1. Samples were obtained on day 5 p.i. (A) Airway inflammation morphology (magnification, ×200). (B) Histological scores from images for panel A. Bars represent means ± SDs from three independent experiments performed in duplicates (*n* = 7). *P* values were calculated based on one-way ANOVA with Bonferroni posttest. ***, *P* < 0.0001; *, *P* < 0.05 for CON versus RSV. ^^^, *P* < 0.0001 for shCON versus shRab5a.

In consideration of the possibility of immune injury after Rab5a knockdown during RSV infection, we assessed airway inflammation at 5 days after RSV infection due to the inflammatory peak at day 5 after RSV infection in our previous studies. The morphological changes (Fig. 7A) and corresponding severity scores (Fig. 7B) showed that the effect of RSV infection in all groups was more severe than noninfection. Among infection groups, knockdown of Rab5a significantly alleviated airway inflammation, suggesting that knockdown of Rab5a decreased RSV replication and reduced airway inflammation.

### Rab5a is upregulated in infants with RSV infection and is associated with disease severity.

**FIG 8:**
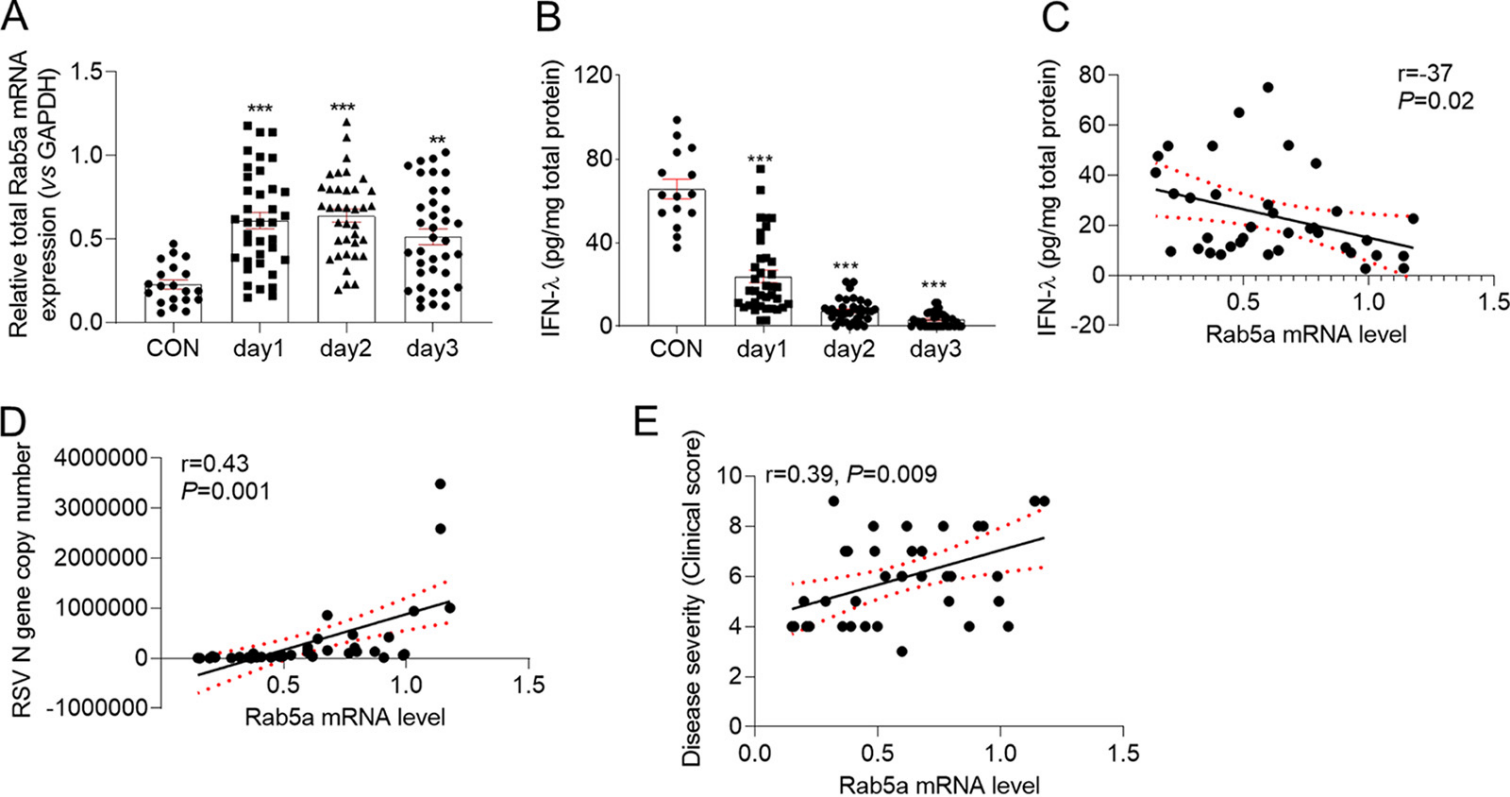
Rab5a is upregulated in RSV-infected infants and is associated with disease severity. Nasopharyngeal aspirate (NPA) samples from 37 RSV-infected infants and 24 uninfected controls were used and processed as described in Materials and Methods. Total mRNA was extracted from the cell pellets. (A) Rab5a mRNA expression in NPA samples. (B) IFN-λ levels in NPA samples. (C) Scatter clustered correlation between Rab5a mRNA level and IFN-λ level. (D) Scatter clustered correlation between Rab5a mRNA level and RSV viral load. (E) Scatter clustered correlation between Rab5a mRNA level and disease severity. Correlations were assessed by Spearman’s tests.

Rab5a is involved in RSV replication, and so next we asked whether it can serve as a therapeutic target in clinical work. We collected 37 nasopharyngeal aspirate (NPA) samples from infants with RSV infection and age-matched controls and then assessed the dynamics of Rab5a mRNA expression level and IFN-λ production in NPA samples. The hospital admission data showed that Rab5a mRNA expression was significantly elevated by the first day of hospital admission for RSV infection, was slightly increased at day 2, and then decreased at day 3 (Fig. 8A). Meanwhile, RSV infection significantly decreased IFN-λ production (Fig. 8B). As the IFN-λ level in some cases was too low to detect at day 2 or day 3, we further correlated the first day’s Rab5a mRNA levels with IFN-λ levels. The correlation data revealed that Rab5a levels negatively correlated with IFN-λ levels (*r* = −0.37, *P* = 0.02) (Fig. 8C). Further analysis showed that the Rab5a mRNA level was positively correlated with viral load (RSV N gene transcriptional level) (*r* = 0.43, *P* = 0.001) (Fig. 8D). These and other studies suggest that RSV viral loads are positively correlated with disease severity. Finally, we compared Rab5a levels with disease severity and found that Rab5a levels positively correlated with disease severity (*r* = 0.39, *P* = 0.009) (Fig. 8E). Together, these clinical and molecular data suggested that Rab5a may affect RSV replication and may serve as a therapeutic target in RSV infection.

## DISCUSSION

Results presented here support an important role of Rab5a in RSV replication. RSV infection increased the amount of Rab5a in airway epithelial cells. The biological effect of this increased Rab5a is to suppress host antiviral immunity. Knockdown of Rab5a gene expression resulted in protection against RSV infection through the activation of IRF1-dependent IFN-λ production. Our study is the first to show that, in airway epithelial cells, (i) RSV infection alters the amount of Rab5a, (ii) Rab5a enhances RSV replication, (iii) the mechanism of this effect of Rab5a involves downregulation of IFN-λ via IRF1, and (iv) knockdown of Rab5a promotes host antiviral immunity and attenuates inflammation of the airway during RSV infection of BALB/c mice. We reiterate that Rab5a may act as a therapeutic target in RSV infection.

Previous studies have demonstrated an effect of Rab5a in diverse virus replication (27–29). Krzyzaniak et al. found that inhibiting the activation of Rab5a attenuates viral titer in RSV infection of HeLa cells (13). Another study, mentioned earlier, found that depletion of Rab5a resulted in decreased RSV replication, and viral binding was not affected when Rab5a action was suppressed (30). We confirmed and extended these studies, as we found that there is no significant difference of viral binding and viral endocytosis between Rab5a-depleted and untreated cells. Together, these results establish that depletion of Rab5a has no effect on RSV binding to airway epithelial cells.

Innate immunity plays an important role in the host's antiviral defense. In our study, we found that knockdown of Rab5a promotes IRF1-dependent IFN-λ production. It is still unknown how Rab5a affects IRF1. Previous studies found that EGFR plays a particularly important role in RSV infection and inflammation (31), and the mechanism is related to IRF1-dependent IFN-λ (21). Multiple studies have shown that EGFR closely interacts with Rab5a. Rab5a is very important for EGFR trafficking, and endogenous EGFRs can partially colocalize with endogenous Rab5 (32). Suppression of Rab5a hampered the degradation of EGFR and its internalization. Furthermore, Rab5a overexpression activates EGFR (33). Indeed, in our study and in those of others, RSV infection activated not only Rab5a but also EGFR (unpublished data). Reciprocally, knockdown of Rab5a decreased the activation of EGFR. The Toll-like receptor 4 (TLR4) signal pathway is also critical for RSV infection, and IRF1 production is regulated by TLR4 signaling (34). TLR4 expression and function on the cell surface are mediated by Rab5a activation (35). From collective evidence, therefore, we predict that Rab5a may affect IRF1 through EGFR or TLR4; however, further investigation is needed to determine how exactly Rab5a influences EGFR and TLR4 during RSV infection.

In our study, we found that, *ex vivo* in cultured A549 cells and *in vivo* in mice, (i) knockdown of Rab5a along with RSV infection further increased IFN-λ expression compared with that from RSV infection alone (Fig. 4E and F) and (ii) knockdown of Rab5a without RSV infection had no effect on IFN-λ production (Fig. 4G and H). However, there were some differences of IFN-λ expression in A549 cells and in mice. Consistent with a previous study (36), we found that *ex vivo*, control cells express small amounts of IFN-λ that are further enhanced by RSV infection, which allow RSV to spread. In contrast, in the infected mice at 12 h and 24 h p.i., control lung cells expressed high levels of IFN-λ, and RSV infection suppressed IFN-λ production. The difference between the *ex vivo* and *in vivo* (in mice) results may be due to several reasons, as follows. (i) A549 is a type II alveolar carcinoma cell line (37), which is oriented toward expression of IFNs and NF-κB proinflammatory cytokines that permit RSV to spread to other cells (36). (ii) In mice, IFN-λ is predominately produced by mucosal epithelial cells that provide critical innate defense functions important for maintaining lung sterility (38). (iii) In mice, the airway environment is more complex, such that besides mucosal epithelial cells, other cell types, e.g., plasmacytoid dendritic cells (pDCs), may also contribute to IFN-λ production, potentially masking the differences of IFN-λ-producing epithelial cells (39).

The nonstructural proteins (NS1 and NS2) of RSV suppress host innate and adaptive immune responses against the virus. Both proteins, individually and in combination, inhibit the type I IFN pathway (40), and the NS1 protein also suppresses IFN-λ production in airway epithelial cells during RSV infection (23). In our study, depletion of Rab5a amplified IFN-λ production in RSV infection; moreover, overexpression of Rab5a increased NS1 mRNA levels (data not shown). Our data, therefore, predict that Rab5a may also promote RSV NS1 production for epithelial cells to suppress antiviral defenses. Further investigations are needed to fully unravel the correlation between Rab5a and RSV NS1.

In conclusion, although limited in scope, our studies have demonstrated that Rab5a plays an important role in RSV infection. We have also discovered a new mechanism by which RSV uses Rab5a to suppress epithelial antiviral immunity, such that silencing of Rab5a results in reduced viral infection. This is a new insight in the role of the cellular factor Rab5a in RSV infection, which can be explored as a therapeutic and druggable target against RSV disease.

## MATERIALS AND METHODS

### Research subjects.

Thirty-seven children with RSV infection, hospitalized from November 2016 to January 2017 in the Department of Respiratory Medicine, Children’s Hospital, Chongqing Medical University, were enrolled in this study. Nasopharyngeal aspirate (NPA) samples were prospectively collected from all subjects within the first day after hospital admission. Immunofluorescence assays were performed to detect the presence of RSV, adenovirus, influenza A and B virus, and parainfluenza virus 1, 2, and 3 in the NPA samples. Infants that carried viruses other than RSV were excluded from the study. Those considered positive for bacterial infection on the basis of published criteria (41) were excluded as well. The control group was selected from infants with no evidence of infection and underwent surgical therapy only to clear secretions from the airway.

### Nasopharyngeal aspirate analysis.

NPA samples were collected and analyzed as previously described (41). In brief, they were collected gently and mixed uniformly. To 0.5 ml of the aspirate, transferred to a new tube, 2 ml of 0.1% dithiothreitol was added. The mixture was vortexed three times, 15 s each, and rocked on a bench rocker for 15 min. The suspension was collected and subsequently centrifuged at 306 × *g* for 10 min. Cell-free supernatants were collected, and aliquots were stored at −80°C. The cell pellet was used for total RNA and protein extraction.

### Reagents, antibodies, and plasmids.

The primary antibodies used in this study include a goat polyclonal antibody to RSV, purchased from Millipore (catalog number [cat. no.] AB1128), rabbit anti-Rab (Rab1a, no.13075; Rab4a, no. 2167; Rab5a, no. 2143; Rab6a, no. 4879; Rab8a, no. 6975; Rab9a, no. 2095; and Rab11a, no. 3539) antibodies, IRF1 (cat. no. 8478), lamin B1 (cat. no. 13435) and glyceraldehyde-3-phosphate dehydrogenase (GAPDH) (cat. no. 5174) monoclonal antibodies, purchased from Cell Signaling Technology (CST), USA. Antibodies against Rab2a (cat. no. PA5-30142) and Rab7a (cat. no. PA5-23138) were purchased from Thermo Fisher, USA. DAPI (4′,6-diamidino-2-phenylindole) was purchased from Sigma-Aldrich, USA. The secondary antibodies used were Alexa Fluor 568/488-conjugated duck anti-goat IgG or anti-rabbit IgG from Beyotime (cat. no. A0428 and A0468), Shanghai, China, and horseradish peroxidase (HRP)-conjugated goat anti-rabbit IgG or anti-mouse IgG from CST (cat. no. 93702). Lipofectamine 2000 (cat. no. 11668500) was purchased from Thermo Fisher.

### Cell culture, virus, and infection.

The human alveolar carcinoma type II-like epithelial cell line A549 (ATCC CCL-185) and human laryngeal cancer epithelial cell line HEp-2 (ATCC CCL-23) were cultured in Dulbecco’s modified Eagle medium (DMEM) supplemented with 10% fetal bovine serum. RSV A2 strain was obtained from ATCC. For all experiments, RSV was grown in HEp-2 cells with 5% fetal bovine serum (FBS) and purified by density gradient as previously described (42). In all experiments where RSV infection was performed in cell culture, a multiplicity of infection (MOI) of 0.8 was used, while mice were intranasally infected with 100 μl RSV of total 1.5 × 10^8^ PFU per ml.

### Animals.

Six- to eight-week-old female BALB/c mice, purchased from the Chongqing Medical University Animal Laboratory, were randomly separated into several groups and were housed in separate filter-top cages under accredited specific-pathogen-free conditions. Mice were sacrificed at designated times after RSV infection. The left lobe of the lung was taken and saved in 4% paraformaldehyde for histopathological studies, while the right lobe was washed with 0.3 ml cold phosphate-buffered saline (PBS) five times to remove the unbound RSV, and the bronchoalveolar lavage fluid was collected. A portion of the right lobe, after the washing step, was stored in 1 ml DMEM per 0.1 g tissue to determine the virus titer in the lung, and another portion was used for protein extraction.

### Virus titration.

At the designated times, the infected cell medium supernatants were collected, and the cells were scraped into the cell culture medium and vortexed three times with glass beads, followed by centrifugation at 1,000 rpm for 5 min. The lungs of the infected mice were homogenized in DMEM on ice and centrifuged at 1,000 rpm for 10 min to obtain the supernatant. All RSV titrations were performed by plaque assay on HEp-2 cells as previously reported (43).

### siRNA transfection.

The Rab (Rab1a, Rab4a, Rab5a, Rab6a, Rab7a, Rab8a, Rab9a, and Rab11a) siRNA sequences were obtained from reference 44 and are shown in Table 1. The siRNA against human Rab2a was purchased from Sigma-Aldrich (cat. no. EMU203561-20UG). Cell lysates were separated by SDS-PAGE and subjected to immunoblotting with rabbit anti-Rabs, rabbit anti-IRF1, or mouse anti-GAPDH (to detect GAPDH as a loading control) antibodies.

**TABLE 1:** Sequences of Rab and IRF1 siRNA and shRNA

Name	Sequence(s) (5′→3′) or source
siRab1a	CAGCAUGAAUCCCGAAUAU; GUAGAACAGUCUUUCAUGA; GUAGAACAGUCUUUCAUGA; UGAGAAGUCCAAUGUUAAA;
siRab2a	Sigma-Aldrich, cat. no. EMU203561-20UG
siRab4a	GAAAGAAUGGGCUCAGGUA; GUUAACAGAUGCCCGAAUG; UUAGAAGCCUCCAGAUUUG; UACAAUGCGCUUACUAAUU
siRab5a	GCAAGCAAGUCCUAACAUU; GGAAGAGGAGUAGACCUUA; AGGAAUCAGUGUUGUAGUA; GAAGAGGAGUAGACCUUAC
siRab6a	GAGAAGAUAUGAUUGACAU; GAGCAACCAGUCAGUGAAG; AAGCAGAGAAGAUAUGAUU; CCAAAGAGCUGAAUGUUAU
siRab7a	GGGAGUUCUGGAGUCGGGAA; CCACAAUAGGAGCUGACUU3’
siRab8a	GAAUUAAACUGCAGAUAUG; GAACAAGUGUGAUGUGAAU; GAACUGGAUUCGCAACAUU; GAAGACCUGUGUCCUGUUC;
siRab9a	duplex CGGCAGGTGTCTACAGAAG
siRab11a	GGAGUAGAGUUUGCAACAA; GUAGGUGCCUUAUUGGUUU; GCAACAAUGUGGUUCCUAU; CAAGAGCGAUAUCGAGCUA.
siCON	ACGUGACACGUUCGGAGAA
siIRF1	duplex UCCCAAGACGUGGAAGGCCAACUUU
shRab5a	CAAGCAGCCATAGTTGTGTAT; CTGGTCAAGAACGGTATCAT
shIRF1	AGGGCTGATCTGGATCAATAA; GATGTTAGCCCGGACACTTTC
shCON	CAACAAGATGAAGAGCACCAA

### Immunoblotting.

Total cell protein (50 μg) as well as whole-lung protein was resolved on 5% to 15% bis-Tris SDS-PAGE gels and then transferred onto polyvinylidene difluoride (PVDF) membranes. The membranes were incubated with primary antibodies against Rab proteins, IRF1, GAPDH, or lamin B1, followed by incubation with appropriate secondary antibodies. The protein bands were visualized with a chemiluminescence kit (Bio-Rad).

### Quantitative PCR.

A549 cells, transfected with plasmids and infected with RSV where mentioned, were harvested, and intracellular RNA was purified at the designated times posttransfection (BioTake). Viral RNA was isolated from the mixture of cells and medium supernatants. The whole-lung tissues of mice were treated the same way as described earlier. RNA (1 μg) was used for first-strand cDNA synthesis, and the cDNA was amplified using the VeriQuest Fast SYBR green qPCR kit (Invitrogen) with the following primers: Rab5a (forward primer, 5′-CAAGAACGATACCATAGCCTAGCAC-3′; reverse primer, 5′-CTTGCCTCTGAAGTTCTTTAACCC-3′); IFNA1 (forward primer, 5′-GTGAGGAAATACTTCCAAAGAATCAC-3′; reverse primer, 5′-TCTCATGATTTCTGCTCTGACAA-3′); IFNB1 (forward primer, 5′-CAGCAATTTTCAGTGTCAGAAGC-3′; reverse primer, 5′-TCATCCTGTCCTTGAGGCAGT-3′); and IFN-λ (forward primer, 5′-CGCCTTGGAAGAGTCACTCA-3′; reverse primer, 5′-GAAGCCTCAGGTCCCAATTC-3′).

RSV copy numbers were quantified with TaqMan qPCR as previously described (45). The PCR cycle conditions were as follows: 50°C for 2 min, 95°C for 10 min, 40 cycles of 95°C for 15 s and 60°C for 30 s. The fold change was obtained using the comparative threshold (2^−ΔΔ^*^CT^*) method using GAPDH as a calibrator.

### Transfection.

All plasmid transfections were performed with a commercial transfection kit (Thermo Fisher Scientific, USA). Cells were seeded on 12-mm coverslips in 24 wells for imaging or in 6-well plates for qPCR or immunoblotting analyses. Experiments were performed at the designated times after transfection. Lentivirus packaged with specific shRNA to knock down Rab5a or with shCON was used to infect the mice intranasally three times before RSV infection.

### shRNA-mediated knockdown.

Lentivirus-packaged nonhuman or mouse shRNA (shCON; 5′-CAACAAGATGAAGAGCACCAA-3′) was used as the negative control. The shRNAs specific for coding sequences are shown in Table 1. Target sequences were cloned into pMKO.1 puro. Viral stocks were produced in HEK293T cells by cotransfecting 2 μg of vesicular stomatitis virus glycoprotein (VSV-G) expression vector, 4 μg of lentiviral packaging construct pCMV-dR8.2 dvpr, and 4 μg of an shRNA expression lentiviral construct using Lipofectamine 2000 (Thermo Fisher) according to the manufacturer’s instructions. The pMKO.1 puro, VSV-G, and pCMV-dR8.2 dvpr expression vectors were obtained from Addgene (plasmid numbers 8452, 12259, and 8455). Virus supernatant from each 10-cm dish after 48 h of transfection were concentrated in 1 ml RPMI 1640 with polyethylene glycol (PEG) 6000. Lentivirus titers were determined by fluorescence-activated cell sorting (FACS) analysis of transduced cells. A total of 10^8^ transducing units of shRNA lentivirus was used to infect each mouse by nasal instillation three times. The knockdown efficiency was confirmed by Western blotting. As shown in Fig. 1F and G, the Rab5a-targeting shRNA decreased Rab5a protein expression by 70% compared to that with shCON.

### Indirect immunofluorescence assays.

RSV-infected A549 cells were fixed with 4% paraformaldehyde for 30 min at room temperature and permeabilized with 0.2% Triton X-100 (Sigma) for an additional 20 min. After being washed and blocked with 2% bovine serum albumin (BSA) for 1 h, the cells were incubated with anti-IRF1 or anti-RSV antibody at 4°C overnight. The cells were then washed three times, followed by incubation with Alexa Fluor 568-conjugated secondary antibodies (Molecular Probes) for 1 h at room temperature. Finally, the cells were visualized and photographed using a Nikon laser confocal microscope, in which the images were acquired with confocal z-section series and were subsequently analyzed with NIS-Elements BR software, version 4.11. For the mouse studies, the lung tissue was sliced (4 μm), mounted on slides, and then treated the same way as cells.

### Syncytium quantification.

Quantification of the RSV syncytia was performed as described by Mehedi et al. (46) with minor modifications. A549 cells were grown on three coverslips per group in 24-well plates, infected with RSV at an MOI of 0.8 where mentioned, and then fixed at 24 h postinfection (p.i.). Three coverslips were imaged and NIS software was used to draw the areas of the syncytia. ImageJ (NIH) was used to confirm the syncytium area.

### Measurement of IFN.

Human-specific enzyme-linked immunosorbent assay (ELISA) kits were used to measure IFN-α, IFN-β, and IFN-λ levels in culture supernatants (BD, USA) and in the bronchoalveolar lavage fluids from RSV-infected BALB/c mice. The procedures were implemented according to the kit instructions.

### Virus binding and entry assay.

A549 cells were seeded on 12-mm coverslips in 24 wells, and siRab5a and siCON were transfected into cells for 36 h. For the binding assay, the transfected (or untransfected control) cells were washed with PBS three times, and then virus (MOI = 10) was added and incubation was performed at 4°C for 10, 30, and 60 min. For the endocytosis assay, the cells were chilled on ice, virus (MOI = 10) was added on ice for 1 h, and then the cells were washed with PBS three times. Complete medium was added and cells transferred to cell incubator for 1 h, and then the plates were transferred onto ice to stop viral entry. Cells were washed three times with PBS to collect the unbound virus and fixed with 4% paraformaldehyde. The cells were then permeabilized with 0.1% Triton X-100 and stained for RSV and F-actin and with DAPI. Images were acquired with a Nikon A1Rsi laser confocal microscope, followed by quantification using the ImageJ software.

### Lung histopathology.

After fixation in 10% (vol/vol) neutral buffered formalin for more than 24 h, the left lung tissues were dehydrated, embedded in paraffin, cut into sections of 4-μm thickness, and stained with hematoxylin and eosin (Sigma-Aldrich). The degree of inflammation of the lung tissue was scored as previously described (45).

### Statistical analysis.

Data analyses were performed using SPSS 19.0 software. One-way analysis of variance (ANOVA) was used to detect the significance of the difference among groups. Unpaired Student’s *t* test was used to detect the significance between two groups. A *P* value of <0.05 was considered significant.

### Ethics statement.

Use of NPA samples from infants was approved by the ethics committee of the Children’s Hospital, Chongqing Medical University (permit number 2015-77). The parents or legal guardians offered written informed consent to participate in the study before the infants were enrolled. All the animal studies were performed in accordance with Chinese Council on Animal Care (license numbers SCXK [Yu] 2012-0001 and SYXK [Yu] 2012-0001).

All procedures were performed in accordance with approved guidelines under the principles of the Declaration of Helsinki.
